# Incorporating the Antioxidant Fullerenol into Calcium Phosphate Bone Cements Increases Cellular Osteogenesis without Compromising Physical Cement Characteristics

**DOI:** 10.1002/adem.202300301

**Published:** 2023-06-24

**Authors:** İlayda Duru, Nisa irem Büyük, Gamze Torun Köse, Dylan Widder Marques, Karina Ann Bruce, John Robert Martin, Duygu Ege

**Affiliations:** Institute of Biomedical Engineering Boğaziçi University Rasathane Street, Üsküdar, İstanbul 34684, Turkey; Department of Genetics and Bioengineering Faculty of Engineering Yeditepe University Ataşehir, İstanbul 34755, Turkey; Department of Genetics and Bioengineering Faculty of Engineering Yeditepe University Ataşehir, İstanbul 34755, Turkey; Department of Biomedical Engineering College of Engineering and Applied Science University of Cincinnati Cincinnati 45236, OH, USA; Department of Biomedical Engineering College of Engineering and Applied Science University of Cincinnati Cincinnati 45236, OH, USA; Department of Biomedical Engineering College of Engineering and Applied Science University of Cincinnati Cincinnati 45236, OH, USA; Institute of Biomedical Engineering Boğaziçi University Rasathane Street, Üsküdar, İstanbul 34684, Turkey

**Keywords:** antioxidants, bones, calcium phosphate cements, Fullerenol, osteogenic differentiations

## Abstract

Herein, fullerenol (Ful), a highly water-soluble derivative of C_60_ fullerene with demonstrated antioxidant activity, is incorporated into calcium phosphate cements (CPCs) to enhance their osteogenic ability. CPCs with added carboxymethyl cellulose/gelatin (CMC/Gel) are doped with biocompatible Ful particles at concentrations of 0.02, 0.04, and 0.1 wt v%^−1^ and evaluated for Ful-mediated mechanical performance, antioxidant activity, and in vitro cellular osteogenesis. CMC/gel cements with the highest Ful concentration decrease setting times due to increased hydrogen bonding from Ful’s hydroxyl groups. In vitro studies of reactive oxygen species (ROS) scavenging with CMC/gel cements demonstrate potent antioxidant activity with Ful incorporation and cement scavenging capacity is highest for 0.02 and 0.04 wt v%^−1^ Ful. In vitro cytotoxicity studies reveal that 0.02 and 0.04 wt v%^−1^ Ful cements also protect cellular viability. Finally, increase of alkaline phosphatase (ALP) activity and expression of runt-related transcription factor 2 (Runx2) in MC3T3-E1 pre-osteoblast cells treated with low-dose Ful cements demonstrate Ful-mediated osteogenic differentiation. These results strongly indicate that the osteogenic abilities of Ful-loaded cements are correlated with their antioxidant activity levels. Overall, this study demonstrates exciting potential of Fullerenol as an antioxidant and proosteogenic additive for improving the performance of calcium phosphate cements in bone reconstruction procedures.

## 1. Introduction

Calcium phosphate cements (CPCs) have been extensively used as clinical bone substitutes for over 20 years. CPCs have a similar structure to natural bone and can easily be applied to osseous defects due to their moldability and low-temperature setting reaction.^[1-4]^ They can produce apatite in physiological conditions and do not harm nearby tissues via heat generation as seen with highly exothermic polymethylmethacrylate (PMMA) cements.^[5,6]^ CPCs are commercially available in a pure form or as composites when combined with various naturally derived polymers such as carboxymethyl cellulose (CMC) and gelatin (gel). When combined with CPCs, CMC improves overall material handling by increasing viscosity^[7,8]^ and enhancing resistance to CPC wash-out.^[8,9]^ Gel was also reported to enhance the mechanical properties of CPCs in many studies.^[10-12]^ Bigi et al.^[10]^ showed that 15 wt% gel increased the compressive strength of apatite CPCs to a value five times greater than unmodified cements. In addition to the contribution of gel on CPC mechanical properties, gel also improves cement biocompatibility.^[10,13]^ Despite the improvement in CPC performance imparted by incorporating these polymers, further enhancements are still needed to heighten CPC osteogenic capabilities^[8,14]^ for deployment in clinical bone augmentation procedures.^[15]^

Many materials have been suggested as cement additives to enhance the osteogenic ability of CPCs. The most heavily investigated candidates are therapeutic growth factor proteins including bone morphogenetic protein-2 (BMP-2)^[16]^ and transforming growth factor-β (TGF-β).^[17]^ However, these recombinant proteins are expensive and difficult to use across an innately heterogenous patient population that often requires variable dosing of these compounds.^[18,19]^ Moreover, disadvantages of BMP-2 use in the clinic such as ectopic bone formation and excessive inflammation^[19,20]^ have been reported. Bioactive ions such as Sr^+2^^[21]^ and Mg^+2^^[22]^ have also been investigated as proosteogenic additives to CPCs with some success. Additionally, recent studies revealed that CPCs loaded with magnetic nanoparticles and primed with electromagnetic fields enhanced osteogenic differentiation of human dental pulp stem cells and rat adipose-derived stem cells.^[14,23]^ Despite the many strategies investigated for improving the osteogenic potential of CPCs, demonstrated efficacy of these new methods in clinical bone reconstruction procedures has remained elusive.

One underexplored strategy for increasing the osteogenic ability of CPCs is the incorporation of antioxidants to reduce local levels of reactive oxygen species (ROS) in the local tissue. Previous studies have shown that elevated levels of ROS cause oxidative stress and can force mesenchymal stem cells, osteoblasts, osteocytes, and MC3T3-E1 preosteoblast cells into apoptosis.^[24-26]^ Moreover, other reports indicate that high ROS concentrations inhibit osteogenic differentiation of mesenchymal stem cells and MC3T3-E1 cells.^[27-29]^ Trolox and selenium, antioxidants that have been extensively studied for bone tissue engineering applications, both enhance cellular osteogenic differentiation and reduce oxidative stress in cells seeded on bone scaffolds.^[29,30]^ These antioxidants were also effective ROS scavengers when incorporated into brushite CPCs,^[31,32]^ though their effects on osteogenic differentiation were not determined with these cements. Therefore, the potential for antioxidants to increase the osteogenic capability of CPCs is still unknown.

Fullerenol (Ful) is a powerful ROS scavenger and a cutting-edge carbon nanomaterial due to its specific physical and chemical features. The electron-deficient positions on the surface of Ful neutralize ROS molecules by efficiently transferring the radicals’ unpaired electrons within the fullerene cage.^[33,34]^ Furthermore, Podolsky et al.^[35]^ suggested that hydroxyl groups on Ful (molecular composition of C_60_(OH)_22–24_) also participate in ROS scavenging, making Ful an even more potent antioxidant than unmodified fullerene. Ful also possesses low cytotoxicity,^[36,37]^ with previous work showing that Ful particles increased the viability of bone marrow macrophage cells^[38]^ and enhanced the proliferation of adipose-derived stem cells.^[37]^ Other studies have reported Ful’s ability to increase osteogenic differentiation of stem cells via upregulation of key osteogenic markers such as alkaline phosphatase (ALP), runt-related transcription factor 2 (Runx2), and osteocalcin (OCN).^[36,39]^ Hence, Ful is a strong candidate to enhance osteogenic properties of CPCs. Therefore, the aim of this study is to enhance the osteogenic ability of CMC/gel-incorporated apatite CPCs by incorporating Ful particles into these materials. For investigating the effect of Ful on the physical characteristics of CPCs, setting time, apatite formation, apatite morphology, compressive strength, and modulus were all investigated. Moreover, the ROS scavenging abilities of Ful-incorporated CPCs were evaluated. Finally, the osteogenic differentiation of pre-osteoblasts treated with Ful-doped CPCs was determined by immunofluorescence (IF) measurements of actin network formation, cellular production of ALP, and polymerase chain reaction (PCR) measurements of osteogenesis-associated gene expression. In short, this work is the first study to incorporate the antioxidant Ful into calcium phosphate cements and demonstrates the exciting potential of this novel nanomaterial as a pro-osteogenic additive.

## 2. Results and Discussion

### 2.1. Characterization of Ball-Milled Powder

Before preparing the cements, the size and crystallinity of cementing powder consisted of tetracalcium phosphate (TTCP) and dicalcium phosphate dihydrate (DCPD) were characterized via scanning electron microscopy (SEM) and X-ray diffraction (XRD). The size and crystallinity of TTCP/DCPD particles before and after ball milling are presented in Figure S1, Supporting Information. Figure S1A,B, Supporting Information, shows that smaller particle sizes in a narrower range were obtained via ball milling. In Figure S1C,D, Supporting Information, the shapes of TTCP and DCPD can be seen; TTCP particles have round edges and smooth surfaces due to their sintering procedure, while DCPD particles are smaller, irregularly shaped, and adhered to the larger TTCP.^[40]^ According to ImageJ analysis (Figure S1E,F, Supporting Information), the average sizes of TTCP and DCPD particles were reduced to 5.1 μm and 162 nm from 18.8 μm and 464 nm via ball milling, respectively. In Figure S1G, Supporting Information, XRD spectra taken before and after ball milling indicate that only DCPD and TTCP phases were present after ball milling.

### 2.2. Physical Characterization of Ful-Incorporated Cements

**Figure 1.**
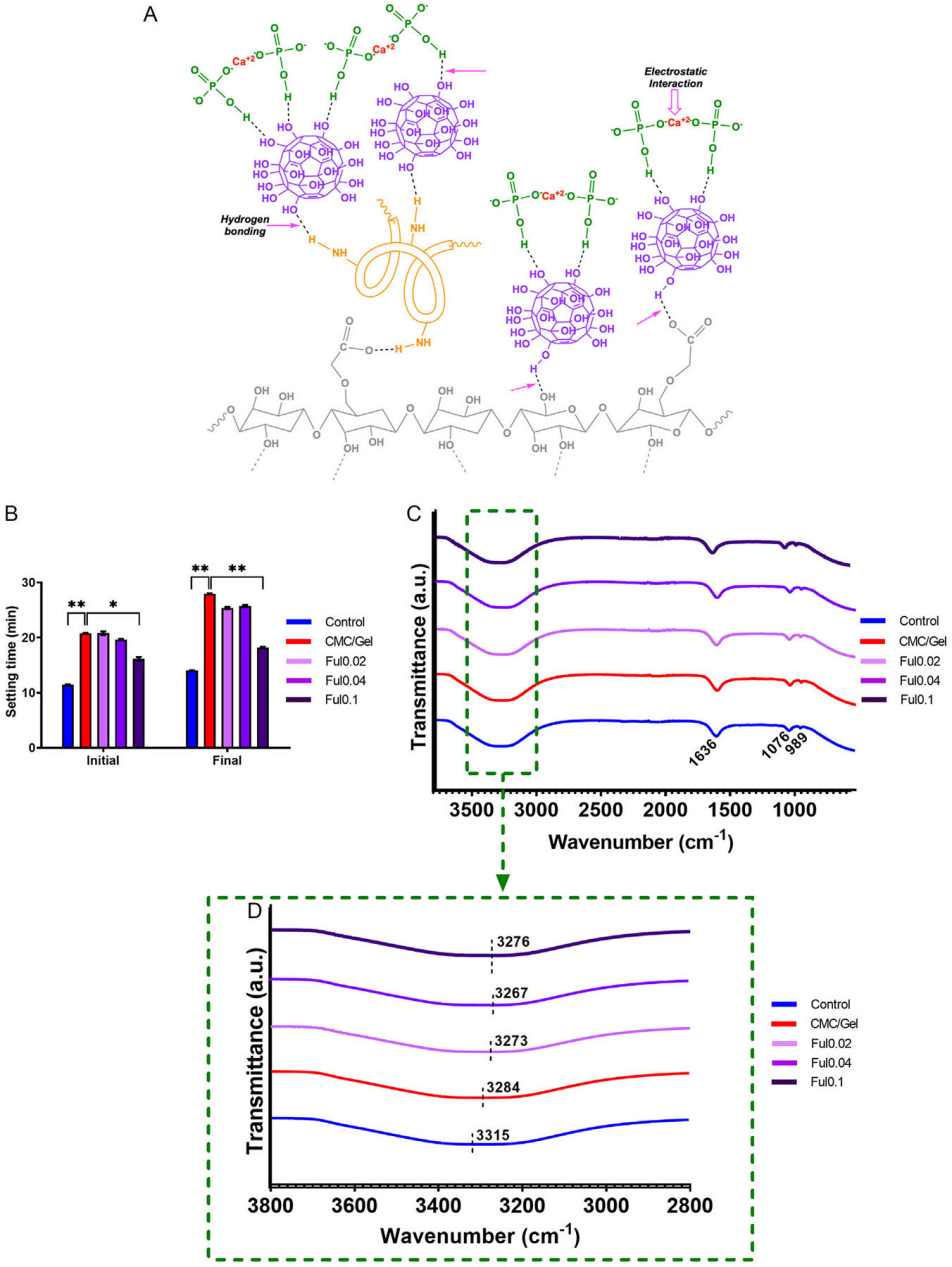
Physical characterization of Ful-incorporated cements. A) Proposed structure and nucleation mechanism during cement hardening. B) Total FTIR spectra of cement liquid phases. C) FTIR spectra of the specific OH band between 2800 and 3800 cm^−1^ of Ful-incorporated cements. D) Initial and final cement setting times (*n* = 6, mean ± SEM, * =*p* < 0.05, ** = *p* < 0.01).

To prepare Ful-incorporated cements, first, 1 wt v%^−1^ CMC and 1.5 wt v%^−1^ Gel were dissolved in hardening agent solution and Ful was incorporated into CMC/gel solution at different concentrations. To determine the molecular interactions of Ful with CMC and gel, Fourier-Transform infrared spectroscopy (FTIR) was conducted. Figure 1 shows FTIR spectra for the liquid phases of control, CMC/gel, Ful0.02, Ful0.04, and Ful0.1 cement formulations. The proposed structure after Ful conjugation to CMC/gel cements is presented in Figure 1A. In Figure 1B, FTIR spectrum of the control cement’s liquid phase shows the characteristic peaks at 989.06, 1076.22, 1636.30, and 3315 cm^−1^. The peaks at 989.06 and 1076.22 cm^−1^ correspond to symmetric stretching vibration and asymmetric stretching vibration of the P─O bond, respectively.^[41,42]^ The peak at 1636.30 cm^−1^ is assigned to the bending vibration of the H─O─H bond, and the peak at 3315.00 cm^−1^ corresponds to the stretching vibration of the OH bond.^[41,43]^

Additions of CMC/gel and/or Ful to the cement liquid phase caused shifting in the OH band as shown in Figure 1C. With the CMC/gel addition, the OH band at 3315.00 cm^−1^ shifted to a lower frequency of 3284.46 cm^−1^ due to stretching vibrations of OH groups in the CMC and stretching vibrations of NH groups in gel.^[44]^ Moreover, 0.02 and 0.04 wt v%^−1^ Ful incorporation to cement liquid phases leads to a further respective downshift to 3273.65 and 3267.49 cm^−1^ due to an increase of OH content and hydrogen bonding in these samples.^[45,46]^

Previous studies also investigated the interaction of Ful particles with amine groups of amino acids and demonstrated that Ful could form hydrogen bonds with these molecules via hydroxyl – amine associations.^[47-49]^ Indeed, Dong et al.^[48]^ observed a shift to a lower frequency in the NH band from an FTIR spectra of phenylalanine/Ful due to hydrogen bonding between amine group of phenylalanine and Ful hydroxyl units. Moreover, hydrogen bonding of antioxidants with carboxyl and hydroxyl groups of polysaccharides was reported by Lombo-Vidal et al.^[50]^ In short, the demonstrated molecular associations between Ful particles and CMC/Gel cement components are well supported from previous studies.

For using moldable materials in orthopedic applications, an initial setting time of 8 min and a final setting time of 15 min are recommended.^[8,51]^ Figure 1D demonstrates the results of setting time measurements. Though baseline CPCs showed initial/final setting times of 11.4/14 min, CMC/Gel addition significantly increased the initial and final setting times to 20.8 and 27.9 min due to immobilization of TTCP and DCPD particles from the viscosity increase.^[8,52]^ The addition of 0.02 and 0.04 wt v%^−1^ Ful did not change initial and final setting times of CMC/gel, though 0.1 wt v%^−1^ Ful significantly decreased the CMC/gel initial setting time to 16.2 min and final setting time to 18.2 min, closer to clinically recommended time frames. These measurements reveal that high concentrations of Ful accelerated the hardening of CMC/Gel cements. This increased cement reactivity is potentially mediated by an increase of the common ion effect via hydrogen bonding of Ful particles to HPO_4_^−2^^[53]^ since the interaction of hydroxyl groups with phosphates has been previously suggested.^[47]^ Therefore, these data indicate that HPO_4_^−2^ sites on Ful0.1 facilitated binding of Ca^+2^ ions to promote apatite nucleation in these Ful-doped cement formulations.

### 2.3. Cement Phase Analysis and pH Measurements in PBS

**Figure 2.**
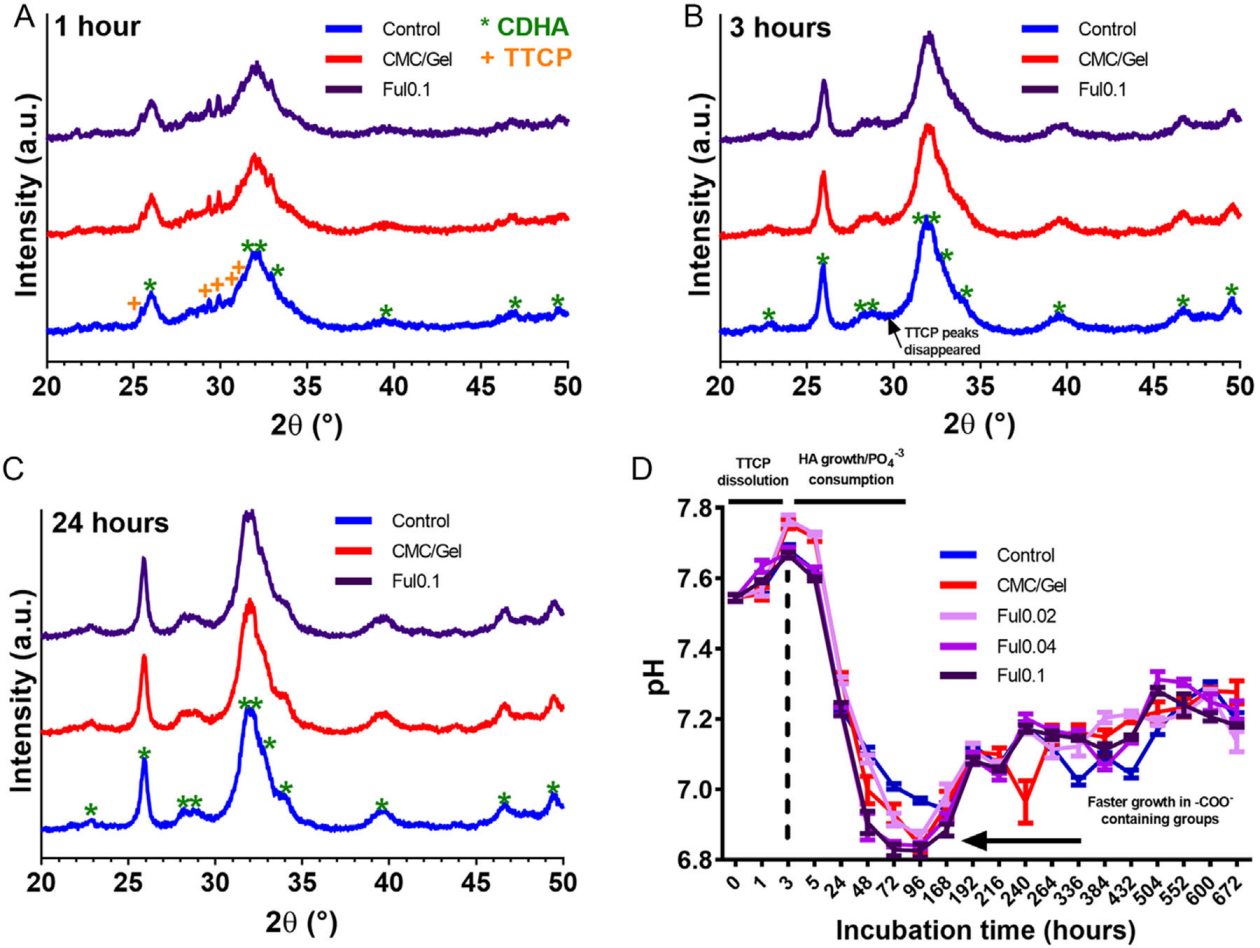
Apatite formation and morphology in Ful-incorporated cements incubated in PBS. A) XRD patterns of cements incubated in PBS for 1 h, B) 3 h, and C) 24 h. D) pH changes of cements incubated in PBS over 672 h (*n* = 5).

Though Ful incorporation into CPC cements decreased their setting time, it did not significantly impact the conversion rate of TTCP and DCPD particles into calcium-deficient hydroxyapatite (CDHA) domains when incubating the cements in PBS, as shown in Figure 2. Figure 2A-C shows XRD spectra at hours 1, 3, and 24 of incubation which reveal the cements’ crystal phases under physiological conditions. It can be deduced that DCPD consumption was completed and apatite started to form in less than 1 h in all cements based on the appearance of characteristic apatite peaks 25.88, 32.05°, and 32.50°.^[54]^ However, there were still residual amounts of TTCP (major peaks at 29.23° and 29.81° as shown in Figure S1G, Supporting Information) at 1 h that were consumed within 3 h so that cements wholly comprised apatite, as shown in Figure 2B. Indeed, the height of the apatite peaks increased between 1 and 24 h to further confirm this conversion. This conversion rate for apatite formation from TTCP and DCPD was one of the fastest reported in the literature as presented in Table S1, Supporting Information,^[54-56]^ and this rapid apatite formation can likely be attributed to the utilization of smaller-sized TTCP and DCPD particles and usage of sodium phosphate solution instead of distilled water.^[57]^ Finally, no differences were observed for the time needed for conversion of TTCP and DCPD to CDHA with Ful incorporation into cements (Figure 2A-C).

Figure 2D shows the pH change of physiological solutions in which the cements were incubated, further confirming the TTCP/DCPD conversion kinetics from the XRD analyses in Figure 2A-C. The pH of all solutions increased in the first 3 h but decreased drastically afterward due to the absence of basic TTCP, consumption of phosphate from the physiological solution, and resulting apatite growth.^[57,58]^ Control cements reached their minimum pH at 168 h (day 7) while CMC/gel, Ful0.02, Ful0.04, and Ful0.1 cements reached their minimum pH at 96 h (day 4). These behaviors are possibly due to greater amount of ─COO^−^ ions from the CMC/gel which can act as nucleation sites for Ca^+2^ ions.^[11,43]^ The minimum pH level for all cement solutions was between 6.8 and 7.0, and after they reached their minimum, pH increased and kept constant between 7.0 and 7.4. Finally, SEM imaging indicated that all cement formulations possessed nanosized platelet-like crystals^[59,60]^ with no obvious differences between groups being observed (Figure S2, Supporting Information).

### 2.4. Cement Phase and Morphological Analysis and pH Measurements in Simulated Body Fluid (SBF)

**Figure 3.**
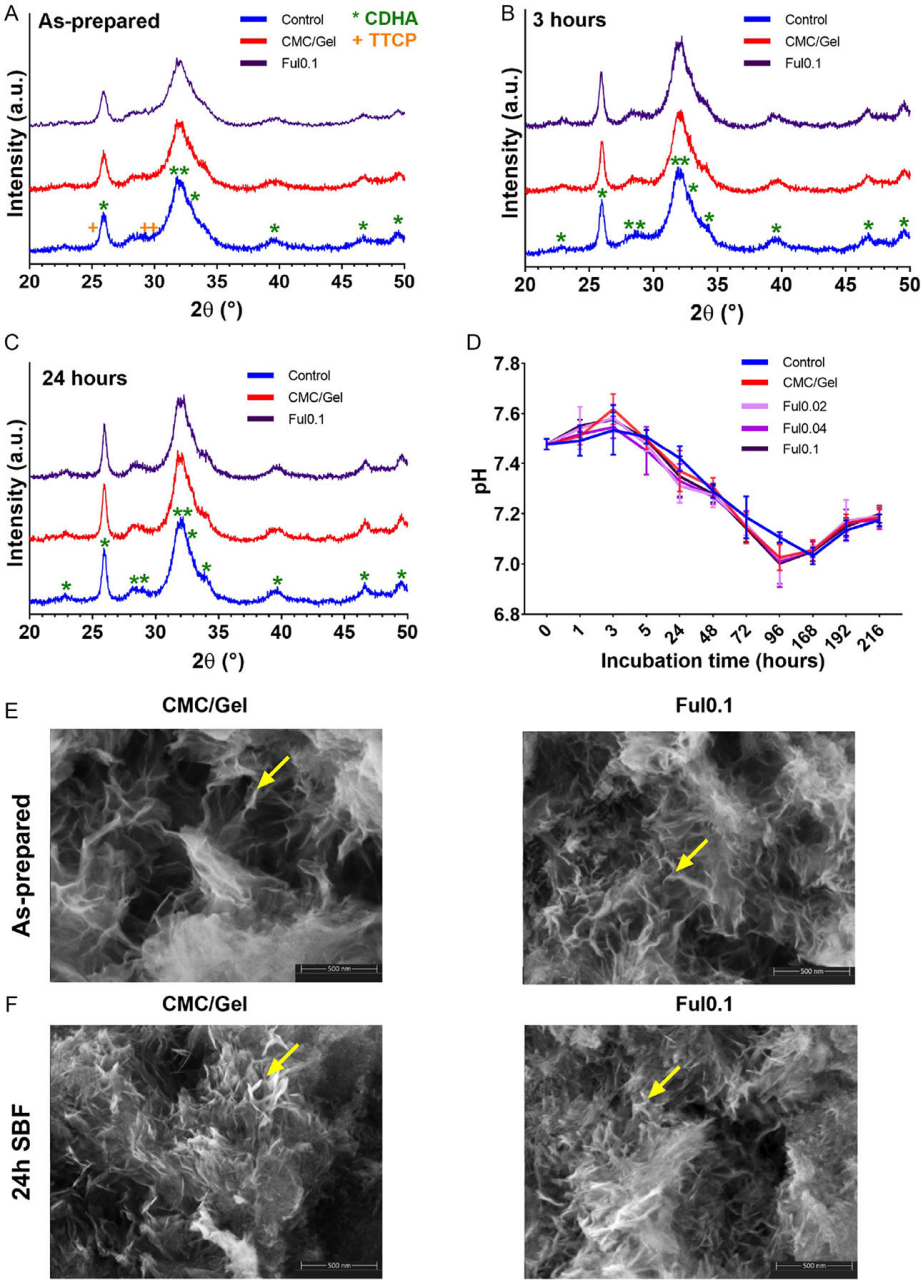
Apatite formation and morphology in Ful-incorporated cements incubated in SBF. A) XRD patterns of as-prepared cements before incubation, or B) cements incubated in SBF for 3 h, and C) 24 h. D) pH changes of cements incubated in SBF over 216 h (*n* = 5). E) SEM micrographs showing the apatite morphology in as-prepared cements and F) SBF-incubated cements for 24 h (Magnification = 160 000×, yellow arrows: apatite cystals).

Phase analyses of cements were also carried out after incubating cement samples in simulated body fluid (SBF) and comparing against as-prepared cements. The XRD spectrum of as-prepared cements (Figure 3A) shows that the crystalline phase of these materials features high levels of apatite even without an SBF incubation. Figure 3B,C shows similar results obtained after the incubation in SBF for 3 or 24 h, demonstrating increasing apatite formation and disappearance of characteristic TTCP peaks. CDHA formation was also determined via energy-dispersive X-ray (EDX) analysis of cements incubated in SBF for 24 h as shown in Figure S3, Supporting Information. The incubated cements achieved Ca/P ratios of 1.40–1.50, which is the Ca/P ratio of CDHA (Figure S3, Supporting Information).^[61]^ Moreover, the pH change of SBF-incubated cements over time (Figure 3D) demonstrated a similar trend with the pH profile in PBS (Figure 2D). Finally, SEM images of cements before incubation in SBF (Figure 3E) and after SBF treatment (Figure 3F) show that all groups included nanosized platelet-like crystals^[59,60]^ indicative of apatite formation (Figure 3E,F). Importantly, as-prepared cements (Figure 3E) did not display obvious differences between PBS-incubated (Figure S2, Supporting Information) or SBF-incubated (Figure 3F) materials

### 2.5. Cement Compression Testing

**Figure 4.**
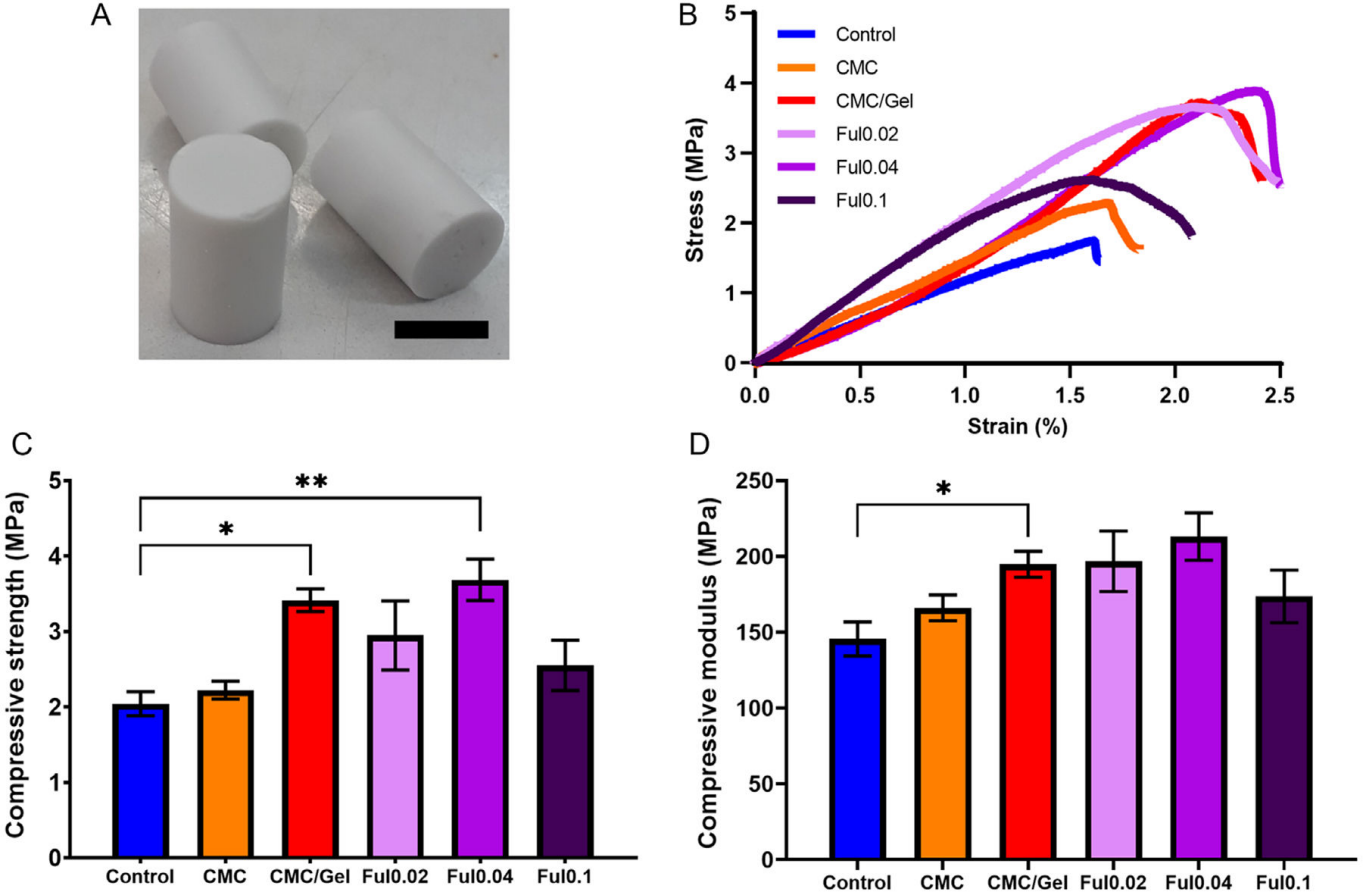
Mechanical analysis of Ful-incorporated cements A) Photograph of cements molded for compression testing (scale bar = 1.25 cm). B) Representative stress–strain curves. C) Compressive strength and D) compressive modulus values of cements (*n* = 5, *for *p* < 0.05, **for *p* < 0.01).

Cancellous bone has compressive strength values between 2 and 16 MPa and compressive moduli in the range of 120–1100 MPa,^[62,63]^ and synthesizing a CPC with proximate mechanical properties is critical for providing mechanical stability with cancellous bone implants.^[64,65]^ As shown in Figure 4, CMC/gel, Ful0.02, Ful0.04, and Ful0.1 cements (model samples shown in Figure 4A) were mechanically characterized through compression testing (representative stress/strain traces shown in Figure 4B). Control cements achieved compressive strength and elastic modulus values of 2.04 and 145.55 MPa, respectively, as shown in Figure 4C,D. In alignment with previous reports,^[9,10,12,13,66]^ solely adding CMC to cements did not alter compressive strength or modulus compared to unmodified control cement samples; however, CMC/gel significantly increased the compressive strength and modulus compared to control samples. Finally, Ful addition did not significantly affect the compressive strength and modulus values of CMC/Gel samples, and all formulations eclipsed the benchmark values of 2 MPa for strength and 150 MPa for modulus. These collective data indicate that Ful-doped cements are mechanically suitable for treatment of nonload-bearing bone reconstructions.

### 2.6. Measurement of In Vitro ROS Scavenging Ability

**Figure 5.**
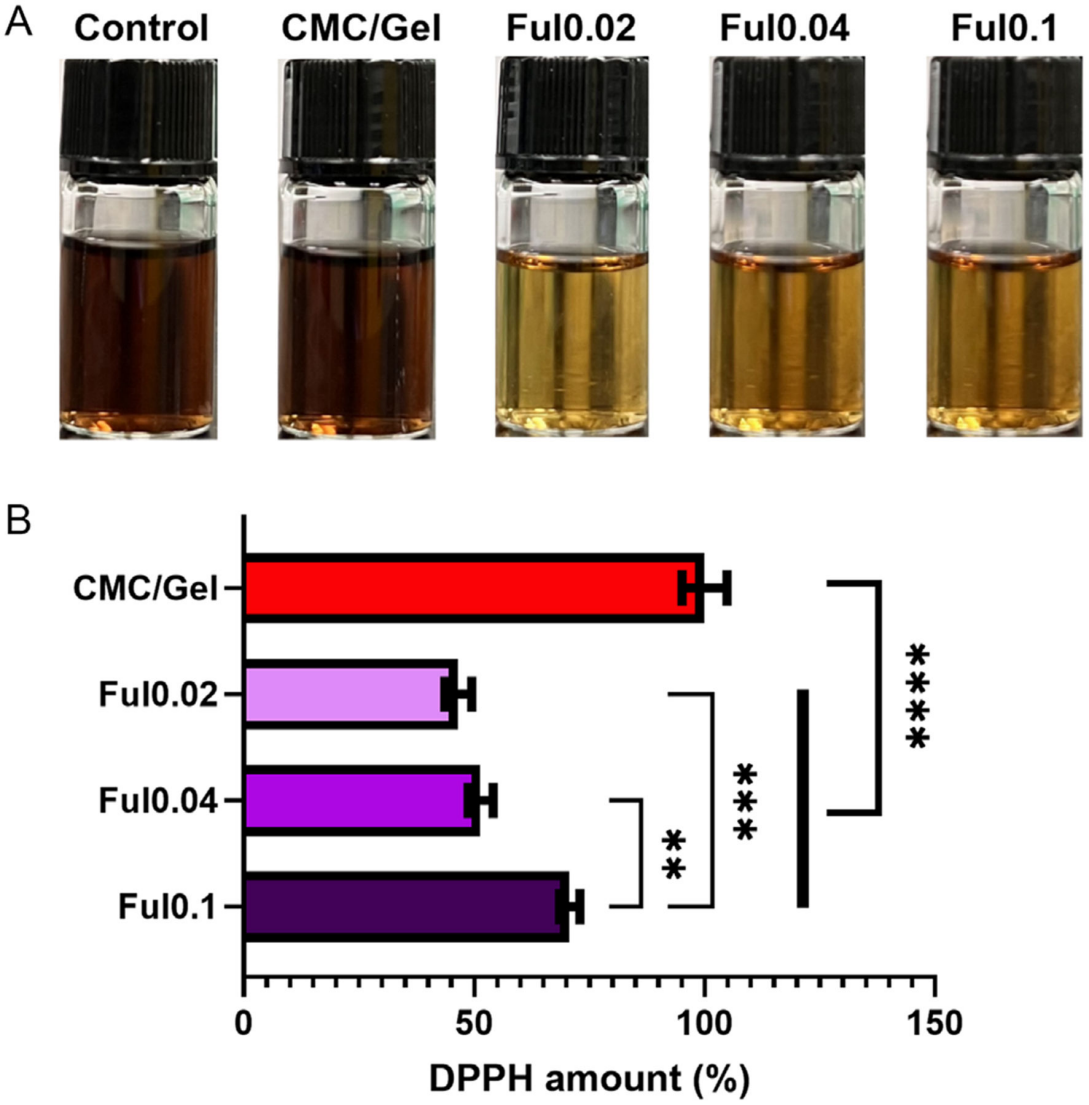
ROS scavenging ability of Ful-incorporated cements. A) Digital photos of DPPH solutions after 24-hour-treatment with cements. B) DPPH inhibition by Ful-incorporated cements (*n* = 5, ** = *p* < 0.01, *** = *p* < 0.001, **** = *p* < 0.0001).

To assess the antioxidant capacity of Ful-loaded CPCs, a DPPH radical inhibition assay was performed on cement samples. Figure 5A demonstrates the color change of DPPH solution after 24 h treatment with Ful-incorporated cements. DPPH’s color change from purple color to yellow is a marker of reduction of DPPH radicals^[67]^ by Ful-incorporated cements, while no change was observed in DPPH solutions treated with control and CMC/gel cements. Quantitative analysis of DPPH activity reduction by Ful-incorporated cements is plotted in Figure 5B, with Ful0.02 and Ful0.04 samples demonstrating 46.58% and 51.41% DPPH inhibition within 24 h. However, DPPH was only inhibited by 29.28% for the Ful0.1 samples potentially due to Ful aggregation, as previously described by Roy et al.^[68]^ for these particles at high concentrations.^[68,69]^ Regarding the significant role of hydroxyl groups both in aggregation and in radical scavenging,^[35]^ it is suggested that Ful particle aggregation diminishes the presentation of active sites on the surface of Ful particles and therefore reduces their interaction with radicals.^[68,69]^

### 2.7. In Vitro Cytotoxicity and Cement Release Kinetics of Ful Particles

**Figure 6.**
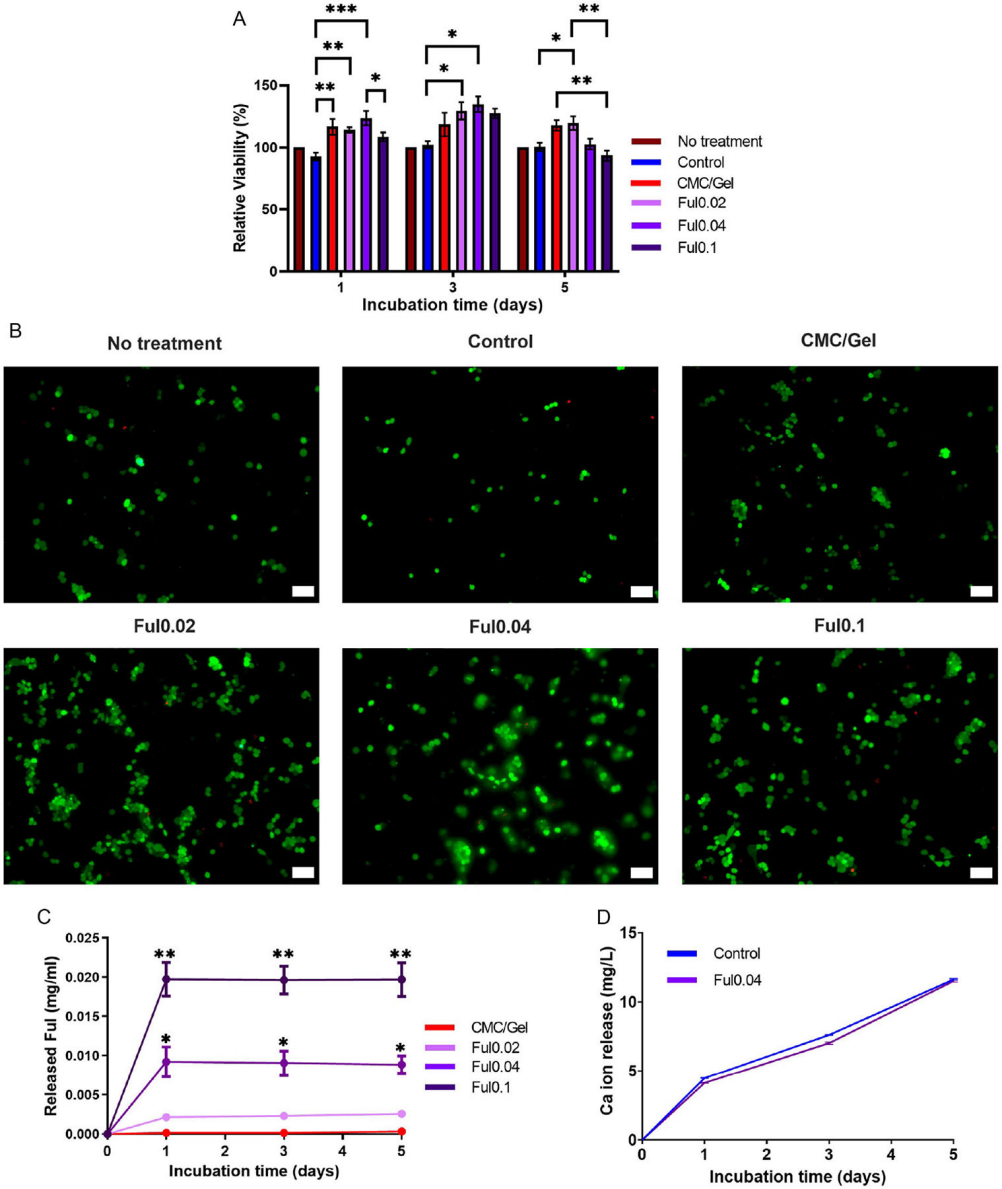
In vitro cytocompatibility of Ful-incorporated cements. A) Viability of L-929 cells cultured in growth media prepared with control, CMC/Gel, Ful0.02, Ful0.04, and Ful0.1 cement extracts collected after 1, 3, and 5 days of incubation (*n* = 5, * = *p* < 0.05, ** = *p* < 0.01, *** = *p* < 0.001). B) Live–dead staining of L-929 cells cultured in growth media prepared with control, CMC/Gel, Ful0.02, Ful0.04, and Ful0.1 cement extracts collected after 3-day incubation (scale bars = 100 μm, green: live cells, red: dead cells). C) In vitro Ful particle release from cements (*n* = 5, *for *p* < 0.05 compared to Ful0.02; **for *p* < 0.01 compared to Ful0.02). D) In vitro Ca ion release from cements (*n* = 4).

In vitro cytotoxicity evaluations of Ful-loaded cement extracts collected from 1, 3, and 5-day incubations were obtained in L-929 cells, as shown in Figure 6A. Though derived from fibroblasts, L-929 cells were chosen for initial cytotoxicity screening of these cements to align with ISO 10 993-5 standard protocols. The day 3 extracts showed that the cell viability increased with medium-dose Ful particles. Moreover, images of extract-treated L-929 with live–dead staining (Figure 6B) agree with the quantitative viability results in Figure 6A as Ful0.02-and Ful0.04-treated cells show more coverage than the other treatment groups.

To be able to understand the reason behind the trend of percent cell viability, both Ful particle and calcium ion release studies were conducted. In vitro release kinetics of Ful particles from Ful-loaded cements are shown in Figure 6C, demonstrating that Ful nearly completely released from the cements within 24 h, though since Ful particles tend to accumulate on the surface of these cements; burst discharge from CPC materials is common.^[31,32]^ Similarly quick release kinetics of antioxidant molecules from CPCs were also reported for trolox^[31]^ and selenium.^[32]^ As expected, the concentration of cumulative Ful released from the cements was proportional to the initial Ful concentration in each sample.

Since the amount of Ful released was the same across the studied time points in Figure 6C, the increase in cell viability on day 3 could be due to the protective effects of Ful against toxicity from excess CPC-released calcium ions, which can be harmful to cells.^[70]^ Excess quantities of calcium ions can compromise cellular viability,^[71]^ and in vitro release kinetics of Ca ions from cement formulations demonstrated that less than 12 mg L^−1^ of free calcium was released from control or Ful0.04 cements over 5 days (Figure 6E) but did increase between the first and fifth days of incubation. Since CPC residues are known to promote in vivo tissue inflammation and increase in vitro ROS levels,^[72-74]^ we concluded that the higher viability levels for cells treated with Ful0.02 and Ful0.04 extracts were likely due to Ful’s antioxidant capacity as similarly demonstrated in other reports.^[37,75,76]^ The cell protective effect of Ful is correlated with its ROS scavenging ability, and as suggested by Hao et al.,^[37]^ Ful increases the expression of MAPK-related proteins p38 and ERK to suppress ROS-induced toxicity. Ful0.1 cements demonstrated lower levels of ROS scavenging than Ful0.02 and Ful0.04 cements.

Finally, all cement fifth-day extracts were still biocompatible; however, the viability percentage of cells incubated in Ful0.1 fifth-day extracts was significantly lower than cells incubated with CMC/gel fifth-day extracts. This result closely correlates with lower ROS scavenging ability of Ful0.1 than Ful0.02 and Ful0.04. Hence, it can be assumed that the high antioxidant capacity of Ful0.02 and Ful0.04 formulations facilitated the increased survival of L-929 cells.

### 2.8. In Vitro Cellular Osteogenic Differentiation Mediated by Ful-Incorporated Cements

**Figure 7.**
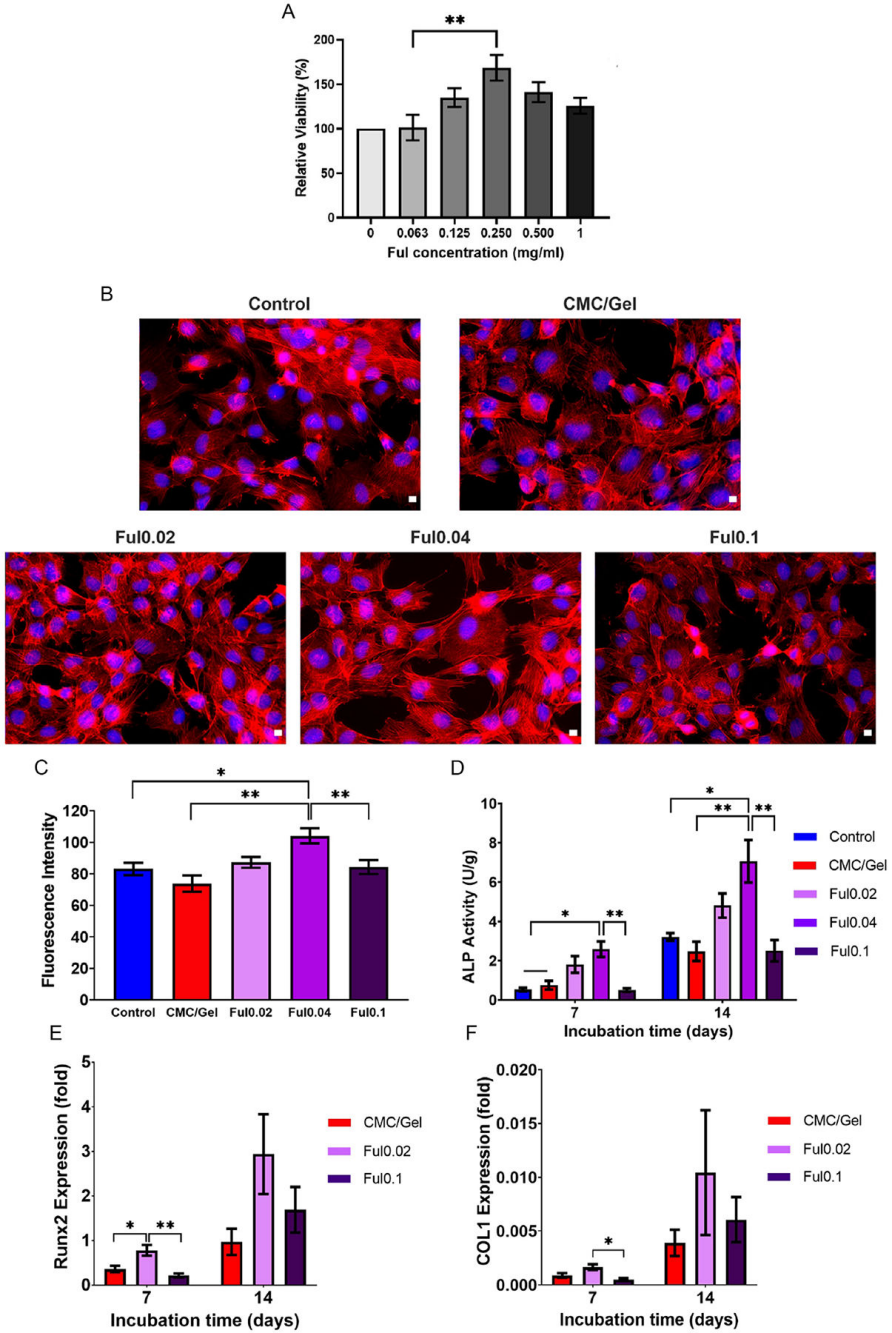
Osteogenic differentiation of MC3T3-E1 cells following treatment with extract media from Ful-incorporated cements. A) Viability of MC3T3-E1 cells after treatment with different concentrations of Ful particles for 24 h. B) Visualization of actin fiber formation following 7-day cement extract treatment via Phalloidin-DAPI staining (scale bars: 10 μm). C) Average fluorescence intensity of actin fibers after differentiation for 7 days. D) ALP activity of cells at days 7 and 14 following treatment with various cement extracts (*n* = 3, * = *p* < 0.05, ** = *p* < 0.01). E) Runx2 expression in cells at days 7 and 14 following treatment with various cement extracts (*n* = 3, * = *p* < 0.05, ** = *p* < 0.01). F) COL1 expression in cells at days 7 and 14 following treatment with various cement extracts (*n* = 3).

To determine Ful’s osteogenic potential when delivered to cells from CMC/Gel cements, IF staining, ALP production measurements, and gene expression studies were conducted with MC3T3-E1 pre-osteoblasts in vitro. The effects of Ful-loaded cements on cellular osteogenic differentiation are shown in Figure 7. First, MC3T3-E1 cells were incubated with varying concentrations of Ful particles to understand Ful’s impact on cell viability (Figure 7A). Crucially, the total dose of Ful released from cements (below 0.025 mg mL^−1^ in Figure 6C) was lower than 0.500 mg mL^−1^ since Ful’s concentration appears to (nonsignificantly) begin reducing cellular viability, as shown in Figure 7A. However, no Ful concentration up to 1 mg mL^−1^ was found to significantly reduce viability of MC3T3-E1 cells over 24 h as in agreement with previous studies listed in Table S2, Supporting Information.^[37,38,77-81]^ When the concentration of Ful reached 0.25 mg mL^−1^, it was discovered that it increased the viability of MC3T3-E1 cells over 24 h, as shown in Figure 7A. Literature also emphasizes that Ful is a highly biocompatible material,^[37,38,77-81]^ and previous assessments with Ful concentrations up to 10 mg mL^−1^ were shown to be non-cytotoxic using human skin fibroblasts,^[77,78]^ murine macrophage cells,^[80]^ and human epidermal keratinocytes.^[79]^

MC3T3-E1 cells induced to differentiate in osteogenic media with respective cement extracts for 7 days were assessed for actin fiber formation (Figure 7B). As shown in these microscopic images, the actin fibers of cells treated with Ful0.04 extracts were denser and more visible than the ones found on other cement groups. The quantitative analysis of actin fluorescence intensity (Figure 7C) also shows that Ful0.04 significantly increased the actin density of MC3T3-E1 cells. F-actin organization/polymerization is a known hallmark of osteogenic differentiation,^[82,83]^ indicating that Ful0.04 extracts had the strongest osteogenic impact on MC3T3-E1 cells compared with the other cement extract treatments. In contrast, the actin density of cells treated with Ful0.1 media was significantly lower than the actin density of Ful0.04-treated cells, indicating that Ful particles are likely aggregating at this higher concentration.^[68,69]^

As shown in Figure 7D, quantification of cellular ALP production following cement extract treatment largely mirrored the results of IF staining. Ful0.04 extract media significantly increased ALP activity in MC3T3-E1 cells compared to the cells treated with control, CMC/gel, and Ful0.1 cement extracts. This significant ALP increase with Ful0.04 treatment was seen at both 7 and 14 days, and ALP production increased over time for all cement extract treatment groups. Moreover, gene expression of the osteogenesis marker Runx2 also increased between 7 and 14 days for all cement treatment groups but tripled with low-dose Ful treatment, as shown in Figure 7E. However, cells treated with the high-dose Ful0.1 cement extracts did not significantly increase Runx2 expression compared to cells treated with CMC/gel extracts (Figure 7E). Finally, a nonsignificant increase of COL1 expression was detected for low-dose Ful cement and it significantly decreased for high-dose Ful cement (Figure 7F).

Previous studies of metabolic changes during the osteogenic differentiation process have conclusively shown that ROS levels are suppressed with antioxidant enzymes during osteogenesis.^[84]^ Despite a somewhat unclear mechanism, numerous in vitro and in vivo studies also suggest that antioxidants can accelerate osteogenic differentiation by reducing ROS concentrations.^[29,36,85,86]^ In addition to the previously suggested antioxidants, the specific effect of Ful on osteogenic differentiation has also been studied.^[36,39]^ Liu et al.^[39]^ showed that Ful particles upregulated Runx2 and OCN levels, while Yang et al.^[36]^ reported that ALP and OCN levels, along with cellular mineralization, were all enhanced following treatment with Ful particles. Moreover, it was suggested that Ful enhanced the expression of FoxO1, which is linked to Runx2, and is responsible for protecting against ROS in bone tissue.^[36]^ Guided by these previous studies, it can reasonably be deduced that the antioxidant capacity of ROS-scavenging particles correlates with their osteogenic ability. This coupling of antioxidant activity and cellular osteogenesis is further supported by the data in this study (Figure 5 and 7), and future analyses with these materials will determine the exact mechanisms relating Ful’s antioxidant activity to bone formation since ROS interacts with several key osteogenic pathways, including Wnt, FOXO, Hedgehog, and MAPK/ERK.^[27,36,39]^ Further in vivo studies with Ful-loaded CPCs in conventional rodent models such as calvarial defects^[21,22]^ or femoral segmental defects^[87]^ are also required to demonstrate the efficacy of Ful in bone healing. However, this study provides strong initial evidence of the benefits Ful can impart to CMC/Gel cements for application in bone reconstruction procedures.

## 3. Conclusion

In this work, the antioxidant molecule Fullerenol was efficiently integrated into a calcium phosphate cementing agent and did not alter apatite formation time, apatite morphology, or compressive mechanical properties. Low Ful concentrations did not impact cement hardening rates, while high Ful concentrations did significantly decrease cement setting times. Crucially, Ful-loaded cements displayed potent antioxidant activity as measured by DPPH radical scavenging assays and correspondingly increased the osteogenic differentiation of MC3T3-E1 pre-osteoblast cells as determined from actin network staining, ALP production measurements, and PCR gene expression analyses. These findings also show that at high concentrations, Ful displayed reduced antioxidant activity (likely due to Ful aggregation) and similarly promoted lower levels of cellular osteogenesis. Overall, these collective data indicate that Ful delivered from bone cements promotes earlier and more potent cellular osteogenic differentiation than unmodified CPCs without compromising physical cement characteristics.

## 4. Experimental Section

### Ball Milling Process and Characterization of Powder:

Tetracalcium phosphate (TTCP, Hitemco Medical, USA) and dicalcium phosphate dihydrate (DCPD, Sigma-Aldrich, United States) were utilized to synthesize calcium-deficient hydroxyapatite (CDHA) cements. A mixture of TTCP and DCPD in equivalent masses was ground and sieved through a 75 μm sieve. TTCP/DCPD particles were ball milled (BM_S38003, MSE Teknoloji) with 3 mm yttrium-stabilized zirconia (MSE Teknoloji) for 48 h in absolute ethanol (Merck, Germany) at 120 rpm.^[88,89]^ The weight ratio of powder/ethanol/ball was 1/2/1.30. Absolute ethanol was selected to leave the powder mixture undissolved, and a milling speed of 120 rpm was chosen from calculating 75% of critical speed.^[90]^

After ball milling, the slurry was dried at 50 °C and grounded into powder. SEM images of particles were taken with a Philips-FEI XL30 in secondary electron mode, and the average particle size was determined via ImageJ. Finally, XRD spectra were obtained using a Rigaku D/MAX-Ultima+/PC equipped with CuKα radiation and step angle of 0.02. The analyzed powder was kept in a vacuum desiccator until its use.

### Preparation of Cements:

The composition of cements is presented in Table 1.

First, the hardening agent solution was prepared. Briefly, a 5.68 wt v%^−1^ Na_2_HPO_4_ (Merck, Germany) solution was produced in distilled water and adjusted to pH of 7.4 via dropwise titration with 1 m HCl (Merck, Germany). Control cements were obtained by mixing a hardening agent solution with ball-milled TTCP/DCPD powder. For CMC/gel solution, 1.5 wt v%^−1^ gel from bovine skin (Sigma-Aldrich, United States) was dissolved in hardening agent solution at 60 °C for 15 min before adding 1 wt v%^−1^ CMC (Sigma-Aldrich, United States). After dissolution of CMC at 90 °C, the solution was cooled at room temperature. CMC/gel cements were prepared via mixing CMC/gel solution with TTCP/DCPD powder as previously described.^[12,91]^

Ful0.02, Ful0.04, and Ful0.1 solutions were obtained by dissolving 0.02, 0.04, or 0.1 wt v%^−1^ C_60_(OH)_n_ · mH_2_O Ful (*n* > 40, *m* > 8, Sigma-Aldrich, United States) in CMC/gel solutions. Ful handling and incorporation into the solutions were all done in an enclosed glovebox or fume hood. Gum-like consistency of the powder/liquid mix was obtained using a powder/liquid weight ratio of 1.25 in all cements.

### Structural Analysis:

Functional groups in the respective cements’ liquid phases were analyzed via FTIR (Nicolet FTIR Instruments, Thermo Fisher Scientific). Spectrum from 4000 cm^−1^ to 550 cm^−1^ was recorded with 32 scans.^[92]^

### Setting Time Measurements:

Setting times of cement samples were measured by the Gilmore test according to standard C266-20 from the American Society for Testing and Materials.^[93]^ Respective specimens were fabricated at 5 mm height and 10 mm diameter, immersed in phosphate buffered saline (PBS, Sigma-Aldrich, United States) and kept at 37 °C and 100% relative humidity. The initial and final setting times were determined using a Gilmore apparatus (Utest) by recording the time at which the light needle (*m* = 113.4 ± 0.5 g, *d* = 2.12 ± 0.05 mm) and heavy needle (*m* = 453.6 ± 0.5 g, *d* = 1.06 ± 0.05 mm) could not make an indentation in the respective sample.

### Phase Analysis and pH Measurements in PBS:

For phase analysis, cements were immersed in PBS at 37 °C for 1, 3, and 24 h. At these time points, the cements were taken from the incubator, removed from PBS, and then immediately frozen at −80 °C. After samples were frozen, they were lyophilized over 24 h before analyzing with XRD. XRD spectra of specimens at 2*θ* = 20°–50° were obtained using a Rigaku D/MAX-Ultima+/PC equipped with CuKα radiation. Step angle was 0.02°.^[94]^

For pH measurements, cement specimens were incubated in PBS at 37 °C and pH of physiological solution were measured via a pH meter (MP225, Mettler Toledo) at specific time points between 1 h and 28 days.^[94]^ During each incubation, PBS was refreshed every 2 days.

### Phase Analysis and pH Measurements in SBF:

SBF was prepared according to the protocol described in Kokubo et al.^[95]^ and cements were incubated in SBF for 3 and 24 h. At these time points, cements were taken from incubator, removed from SBF, and taken to −80 °C freezer with as-prepared cements. Subsequently, they were lyophilized for 24 h. XRD spectra of the cements were obtained via Bruker D8 Advance XRD. For pH measurements, cements were incubated in SBF and measured for pH changes over time using a pH meter (MP225, Mettler Toledo) at specific time points between 1 h and 14 days. SBF was refreshed every 2 days.

### Imaging of Surface Morphology and Apatite Formation:

For imaging of surface morphology and apatite formation, cement samples were kept in either PBS or SBF at 37 °C for 24 h.^[96]^ After 24 h, the aqueous media were removed and specimens were lyophilized. Lyophilized samples were sputter coated with platinum, and cross-sectional images of fractured surfaces were taken with a Philips-FEI XL30 SEM in secondary electron mode in 100x–100,000x magnification. Cross-sectional images of as-prepared and incubated cements were collected with a Thermo Scientific Quatro S Environmental SEM in secondary-electron mode via Everhart–Thornley detector (ETD) detector to increase image magnification to 160 000× without the need for sample sputter coating. For elemental analysis, EDX was conducted on the same device using a gaseous secondary electron detector (GSED) detector to determine cements’ elemental Ca/P ratio.

### Compression Test:

For the mechanical analysis of the cements, samples were molded with 10 mm diameter and 20 mm length. Before testing, the cements were incubated at 37 °C for 24 h in PBS before confirming height and diameter values for the respective samples. Compression tests were performed on wet samples using a Zwick roell z100 testing system with a 100 kN load cell and a crosshead speed of 0.5 mm min^−1^.^[97]^ Compressive strength and elastic modulus values were calculated from the processed stress/strain data.

### In Vitro ROS Scavenging Ability Test: In Vitro:

ROS scavenging potential of Ful-containing cements and CMC/Gel were determined using a 1,1-Diphenyl-2-picrylhydrazine (DPPH) assay.^[98,99]^ DPPH solutions was prepared by dissolving DPPH (31.62 mg, Sigma-Aldrich, United States) in 80:20 v/v% solution of ethanol (400 mL, Thermo Fisher Scientific, United States) and deionized water. Respective cement samples (10 mg) were placed into DPPH solution (2 mL) and shook at 37 °C in the dark. At 1, 12, and 24 h, samples of DPPH (100 μL) were removed and absorbance was measured on a Tecan MPlex microplate reader at 517 nm. Absorbance values of DPPH solutions incubated with Ful containing cements ($A_{\text{Ful}}$) were compared to absorbance readings of DPPH solution incubated with CMC/gel cement as shown in Equation (1) below.


(1) $$\%\;\text{DPPH Amount}=1-\left(\frac{A_{\text{Ful}}-A_{\text{CMC}∕\text{Gel}}}{A_{\text{CMC}∕\text{Gel}}}\right)\times 100$$

### In Vitro Ful Release from CPCs:

For determining the amount of Ful released from cements, Ful particles were labeled with fluorescein 5-isothiocyanate (FITC, Thermo Fisher Scientific, United States) via an isothiocyanate-hydroxyl conjugation. First, Ful (1 mg) was dissolved in anhydrous dimethyl sulfoxide (2 mL, Sigma, Germany). Then, FITC (3 mg) was added into solution and stirred for 16 h at room temperature in the dark. To extract the FITC-labeled Ful, acetone (13 mL, Thermo Fisher Scientific, United States) was added to the reaction solution to precipitate out Ful-FITC while leaving unreacted FITC in the supernatant. The precipitated reaction mix was then spun down at 3000×g for 5 min before removing the supernatant and air drying the remaining Ful-FITC. As described above, cements were then prepared with the Ful-FITC along with control and CMC/gel cements.

Cement samples (*n* = 3 samples per group, 30 mg) were placed into individual wells in 24-well tissue culture plates containing PBS (1 mL). Then, well plates were put on a shaker and kept in the dark. On days 1, 3, and 5, the fluorescence emissions of releaseate media were measured at FITC’s excitation wavelength at 495 nm and emission wavelength at 520 nm via microplate reader.^[100]^ Fluorescence of pure PBS was subtracted from the releaseate fluorescence readings. Finally, Ful-FITC release kinetics were determined using a standard curve prepared from FITC solutions in serial dilutions of PBS.

### In Vitro Cell Culture Studies:

Murine L-929 fibroblasts (American Type Culture Collection (ATCC), United States) and murine MC3T3-E1 pre-osteoblasts (Subclone 4, ATCC, United States) were the two immortalized cell lines used for in vitro biological evaluations. L-929s were cultured in Dulbecco’s modified Eagle’s Medium low glucose (Gibco, United States) supplemented with 10% fetal bovine serum (Gibco, United States) and 1% penicillin-streptomycin (Gibco, United States). MC3T3-E1 cells were cultured in either growth medium (alpha-MEM without ascorbic acid (Gibco, United States), 10% fetal bovine serum, and 1% penicillin-streptomycin) or osteogenic induction medium (growth medium supplemented with 10^−8^ m dexamethasone (Thermo Fisher Scientific, United States), 0.01 mol L^−1^ L-ascorbic acid (Thermo Fisher Scientific, United States), and 50 mg mL^−1^ β-glycerophosphate (Thermo Fisher Scientific, United States)). All cells were maintained in a 95% humidified incubator with 5% CO_2_ at 37 °C.

### In Vitro Cytotoxicity Evaluation:

A cytotoxicity test was conducted according to ISO 10 993-5.^[101]^ Cement samples (30 mg) were immersed in sterile PBS (1 mL, Sigma-Aldrich, United States), and the extracts were aspirated on days 1, 3, and 5. Then, the extracts were sterile filtered through 0.22 μm polyvinylidene fluoride (PVDF) hydrophilic membranes and combined with growth medium in a 1:10 ratio.

When cultured L-929 cells reached confluency, they were seeded in a 96-well plate at a concentration of 5 × 10^3^ cells/well.^[102]^ After overnight incubation, old media were removed, and cells were treated with PBS (100 μL) with or without the respective cement extracts for 24 h. Metabolic activity was determined via the MTS assay (Promega, United States). The absorbance was measured at 490 nm using a microplate reader (Varioskan Lux, Thermo Fisher Scientific). The results were normalized to the absorbances of the nontreated group.

### Live–Dead Staining:

Live–dead staining was performed on the cells treated with day three cement extracts. First, the staining solution was prepared by adding calcein (0.5 μL, Thermo Fisher Scientific, United States) and ethidium bromide (2 μL, Thermo Fisher Scientific, United States) to 1 mL D-PBS (Gibco, United States). Then, the media on the cells was removed from the wells, cells were rinsed with D-PBS, and staining solution was added to each cell well before incubating at 37°C for 45 min. Finally, the images were obtained via fluorescence microscopy using FITC and Texas Red imaging channels (Axiovert A1, Zeiss).^[103]^

### Cell Viability Test with Ful Particles:

MC3T3-E1 cells were cultured to confluency before seeding at a density of 5000 cells/well in a 96-well plate for 24 h. Subsequently, the cells were treated with Ful 100 μL in cell culture media in serial dilutions including 1000, 500, 250, 125, 62.5, and 0 μg mL^−1^ for 24 h.^[37]^ Finally, the solutions in each well were aspirated, and the CellTiterGlo (Promega, United States) solution was added. After incubation for 10 min at room temperature, the bioluminescence of the wells was measured at 515 nm with a microplate reader (Tecan MPlex). Luminescence values for Ful-treated wells were normalized to the nontreated group.

### In Vitro Osteogenic Differentiation Studies:

For osteogenic differentiation studies, cement formulations were incubated in MC3T3-E1 growth medium for 1 week before extracts were collected.^[21]^ To prepare the osteoblastic induction medium for each cement, these extracts were filtered through sterile 0.22 μm PVDF filters and combined with the osteoblastic induction medium in a 1:1 ratio. Cells were cultured in growth medium for 24 h. On the second day, half the medium in each well was removed before adding 1 mL of respective cement extract media samples. The osteoblastic induction medium was changed every 2 or 3 days.

### Immunofluorescence Staining:

Cytoskeletal differences in treated MC3T3-E1 cells were observed on day 7 post-treatment. The culture medium was removed, cells were washed with PBS, and the cells were fixed with 3.7% formaldehyde (Riedel-De-Haën, Germany) for 30 min at room temperature. Fixed cells were permeabilized for 5 min with 0.1% Triton-X 100 (Biobasic, Canada) before being incubated in blocking solution for 10 min. Cell cytoskeletal actin was visualized by treating the cells with Alexa Fluor 594 phalloidin (Thermo Fisher Scientific, United States) for 60 min. Subsequently, the samples were rinsed with PBS to remove unbound conjugates. Moreover, cell nuclei were stained with 4′,6-diamidino-2-phenylindole (DAPI, Sigma, Germany) for 15 min. Finally, the samples were rinsed thoroughly with PBS before adding Prolong Diamond Antifade Mountant (Thermo Fisher Scientific, United States) and imaged under the fluorescence microscope (VertA1, Zeiss, Germany) using the TRITC and DAPI channels. Finally, the fluorescence intensity of cells was measured via ImageJ.

### Alkaline Phosphatase (ALP) Activity Test:

For the ALP activity test, MC3T3-E1 cells were seeded at a density of 10 000 cells cm^−2^ in 6-well plates (2 mL media per well) and cultured in osteoblastic induction medium for either 7 or 14 days. At these time points, medium from the cells was first aspirated before briefly rinsing with D-PBS and trypsinizing. The trypsinized cells were added to individual microcentrifuge tubes (1.5 mL) and lysed in cold D-PBS via vortexing. Lysates were processed to measure ALP activity according to the manufacturer’s instructions (Abcam, United States). Briefly, the lysates were resuspended in assay buffer (500 μL) and centrifuged at 4 °C for 15 min at 16 000 rpm. Precipitations were removed, and supernatants were transferred to a new tube and kept cold during the following steps.

To measure ALP activity, 80 μL supernatant samples were put into wells in 96-well tissue culture plates in serial dilutions before adding p-nitrophenyl phosphate solution (50 μL) to each well.^[104]^ After incubation for 60 min in the dark, NaOH solution (20 μL) was added, and absorbance at 405 nm was measured via a microplate reader (Tecan MPlex). The absorbance of the background was also considered and subtracted from the absorbance of the sample wells. Finally, the p-nitrophenol (pNP) concentration of each well was calculated using the prepared standard curve, and ALP activity was calculated as shown below.

(2) ALP activity(U∕mL)=(A∕V)∕T

where A is the amount of pNP generated in samples calculated from the standard curve (μmol), V is the volume of sample added in assay well (mL), T is the reaction time (minutes), and units are glycine units. A Bradford assay was also utilized to calculate protein amounts for normalizing ALP activity. Briefly, Coomassie reagent (250 μL, Bioworld, United States) was added to supernatant (5 μL) and incubated for 10 min at room temperature. Finally, absorbance was measured at 595 nm using a microplate reader (Tecan MPlex), and protein amount was calculated according to the prepared standard curve calculated with solutions of bovine serum albumin protein.

### PCR:

MC3T3-E1 cells were seeded in a 12-well plate at 20 000 cells per well and incubated for 24 h to allow cells to adhere. After incubation, cells were treated with 1 mL of a 1:1 volumetric mix of osteoblastic induction medium and filtered extracts from respective cement formations (*n* = 3 wells per treatment). Cells were allowed to differentiate for 7 and 14 days, with media being replaced with fresh osteogenic media on days 4, 7, and 10. On days 7 and 14, cells were washed and lysed before extracting and purifying RNA using a PureLink RNA Mini Kit (Invitrogen, Waltham, MA), following the manufacturer’s protocol. Extracted RNA was quantified for quality and concentration using a NanoQuant plate on a Tecan MPlex microplate reader. cDNA was synthesized using an iScript Reverse Transcription Supermix for RT-qPCR (Bio-Rad, Hercules, CA). Quantitative real-time PCR (qRT-PCR) was performed using iTaq Universal SYBR Green Supermix (Bio-Rad, Hercules, CA). Relative expression levels of runt-related transcription factor 2 (RUNX2) and Collagen Type 1 (COL1) were normalized to glyceraldehyde 3-phosphate dehydrogenase (GAPDH) using the ΔΔCt method. Primer sequences are given in Table 2.

### Statistical Analysis:

Unless otherwise stated, all experiments employed at least *n* = 3 samples per group. Results were reported as mean±standard error (SEM) and *p* < 0.05 was considered statistically significant. Statistical analyses were performed using IBM Statistics 25. Normality of data were confirmed via Shapiro-Wilk testing. For ALP activity on 14^th^ day and setting time analysis, the significance of difference was determined via Kruskal–Wallis with Dunn’s test for pairwise comparisons. For the other analyses, one-way ANOVA followed by Tukey posthoc testing was utilized. GraphPad Prism 9 was utilized to plot the results and single asterisk (*), double asterisk (**), triple asterisk (***), and quadruple asterisk (****) were used to represent *p* < 0.05, *p* < 0.01, *p* < 0.001, and *p* < 0.0001 respectively.

## Supplementary Material

supplement

## Figures and Tables

**Table 1.** Composition of cements.

	Liquid phase [wt v%^−1^]			Powder/Liquid [wt/wt]
	CMC	Gel	Ful	
Control	0	0	0	1.25
CMC/Gel	1	1.5	0	1.25
Ful0.02	1	1.5	0.02	1.25
Ful0.04	1	1.5	0.04	1.25
Ful0.1	1	1.5	0.1	1.25

**Table 2.** Primer sequences used in qRT-PCR.

Gene	Forward Primer	Reverse Primer
RUNX2	5′-CACTGGCGGTGCAACAAGA-3 ′	5′-TTTCATAACAGCGGAGGCATTTC-3′
COL1	5′-CCTGAGTCAGCAGATTGAGAACA-3′	5′-CCAGTACTCTCCGCTCTTCCA-3′
GAPDH	5′-GGTGAAGGTCGGTGTGAACG-3′	5′-CTCGCTCCTGGAAGATCGTG-3′

## Data Availability

The data that support the findings of this study are available from the corresponding author upon reasonable request.
