# Supplementary material for: Incorporating the Antioxidant Fullerenol into Calcium Phosphate Bone Cements Increases Cellular Osteogenesis without Compromising Physical Cement Characteristics
Source: Adv Eng Mater. Author manuscript; Available in PMC 2023 Nov 17. (PMC10656051; doi:10.1002/adem.202300301)
Supplement: supplement [file NIHMS1915638-supplement-supplement.pdf]

Supporting Information

Incorporating the antioxidant Fullerenol into calcium phosphate bone cements increases cellular osteogenesis without compromising physical cement characteristics

İlayda Duru[1], Nisa İrem Büyük[2], Gamze Torun Köse[2], Dylan Widder Marques[3], Karina Ann Bruce[3], John Robert Martin[3], Duygu Ege[1*]*

Figure S1 shows the size and crystallinity of TTCP and DCPD powder before and after ball milling.

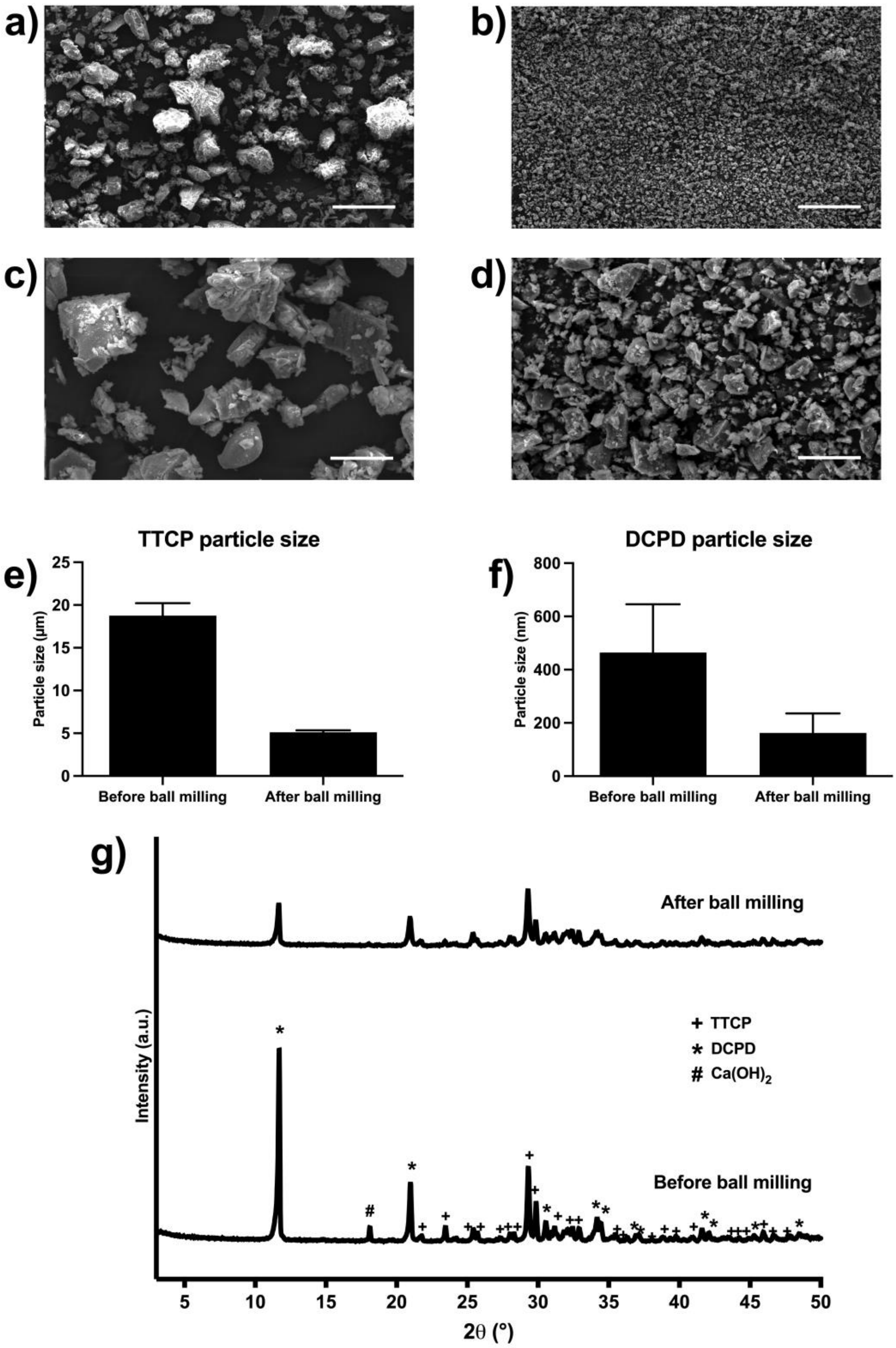

Figure S1 Change of particle size and crystallinity via ball milling A) SEM image of TTCP/DCPD particles before ball milling (Scale bar=50 μm) B) SEM image of TTCP/DCPD particles after ball milling (Scale bar=50 μm) C) SEM image of TTCP/DCPD particles before ball milling with higher magnification (Scale bar=10 μm) D) SEM image of TTCP/DCPD particles after ball milling with higher magnification (Scale

bar=10 µm) E) Change of TTCP particle size via ball milling F) Change of DCPD particle size via ball milling G) X-ray diffraction (XRD) spectrums of TTCP/DCPD particles before and after ball milling

Figure S2 demonstrates the apatite morphology in cements incubated in PBS for 24 hours.

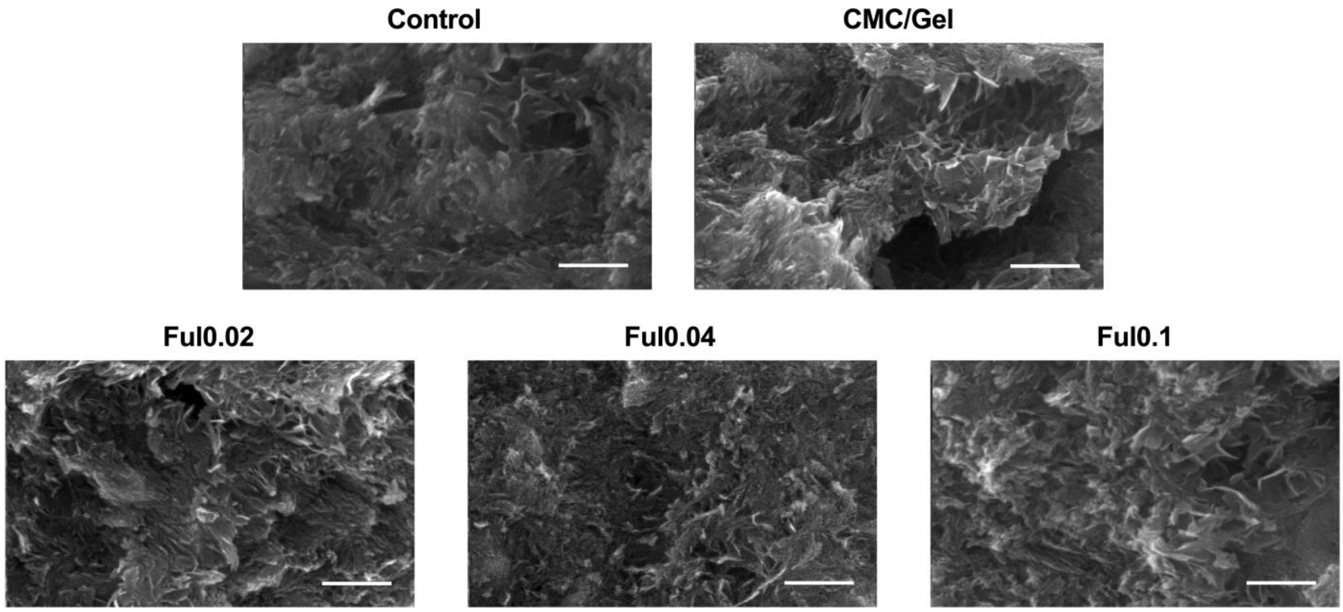

Figure S2 Apatite morphology in cements incubated in PBS

Figure S3 demonstrates the EDX analysis on cements incubated in SBF for 24 hours.

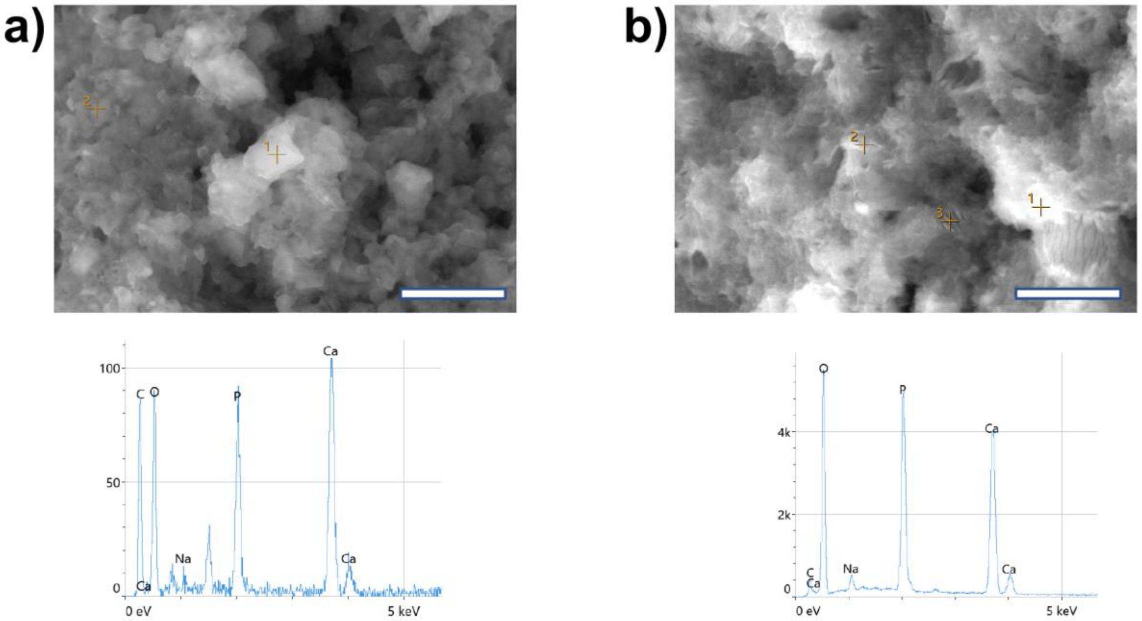

a)

	Ca	P	Ca/P
1	5.6	3.8	1.47
2	6.2	4.0	1.55
Avg			1.51

b)

	Ca	P	Ca/P
1	14.9	10.6	1.41
2	15.3	11	1.39
3	20.7	13.8	1.5
Avg			1.43

Figure S3 EDX analysis of a) CMC/Gel cements and b) Ful0.1 cements after incubation in SBF for 24 hours

Table S1. Apatite formation time of TTCP and DCPD particles possessing different sizes in different liquid phases

	**Particle size of TTCP and DCPD (μ)**	**Liquid phase**	**Conversion of TTCP and DCPD to CDHA (hours)**
This study	**TTCP:** 5.1<br>**DCPD:** 0.162	Sodium phosphate	3
Neira et al.[58]	**TTCP:** 1.5<br>**DCPD:** 1.1	Distilled water	6

Burguera et al.[43][59]	**TTCP:** 17<br>**DCPD:** 1.7	Sodium phosphate	>24

Table S2. Related articles studied Ful emphasizing binding mechanism with molecules, ROS scavenging mechanism and the effect of ROS scavenging mechanism on tissue formation

Ful or Ful composite (Studied concentration range)	ROS scavenging ability and interactions	Cytotoxicity/Pro-osteogenic ability
Ful particles[36][37]	-Ful catches free radicals via the electron-deficient positions on its surface and scavenges them via transferring them to fullerene cage.	N/A
Ful particles[38]	-Hydroxyl groups of Ful takes part in DPPH scavenging.	N/A
Ful particles[73][74] (0.1-50 μg/mL)	-Reduction in ROS scavenging ability of Ful at high concentration is due to aggregation of Ful at high concentration.	N/A
Ful particles[52]	-The amount of hydroxyl group can be significant to determine the binding mechanism of Ful with protein. Hydrogen bonding is common between a protein and a Ful with high amount of hydroxyl group while hydrophobic interaction can be considered between aromatic chains of Ful and hydrophobic surface of protein.	N/A
Ful particles[51]	-Hydrogen bonding between Ful and phenylalanine caused a shift to lower	N/A

	frequency at OH band in FTIR spectrum of phenylalanine.	
Ful particles[50]	-Ful binds to phosphate backbone of DNA.	N/A
Ful particles[79] (0.05-0.15 mg/mL)	N/A	-Ful with concentration up to 0.15 mg/mL is non-toxic to blood mononuclear cells.
Ful particles[77] (0.0005-42.5 μg/mL)	N/A	-Ful with concentration up to 0.04 mg/mL is non-toxic to human epidermal keratinocytes.
Ful particles[76] (1.00-10.00 mg/mL)	N/A	-Ful with concentration up to 10 mg/mL is non-toxic to human skin fibroblasts.
Ful/gentamicin[75] (0.058-29.25 μg/mL)	N/A	-Ful with concentration up to 0.03 mg/mL is non-toxic to human skin fibroblasts.<br>-Ful decreased toxicity sourced from gentamicin via ROS scavenging.
Ful/alginate[40] (10-600 μg/mL)	N/A	-Ful with concentration over 100 μg/mL enhanced the survival of adipose-derived stem cells on alginate hydrogel via activating ERK and p38 pathways, inhibiting JNK pathways and suppressing ROS.
Ful particles[41] (1-100 μM)	N/A	-Ful with a concentration above 1 μM enhanced the viability of bone marrow macrophage cells.
Ful particles[39] (0.1-10 μM)	N/A	-Ful with concentration up to 10 μM is non-toxic to adipose derived stem cells.

		-1.0 μM Ful increased ALP and Runx2 expression and 0.1 μM Ful enhanced OCN expression and mineralization.<br>-The increase of osteogenic differentiation with Ful addition is related to the enhanced expression of FoxO1 which is a transcription factor in defense mechanism against ROS and linked to Runx2.
Ful particles[42]<br>(0.1 and 1.0 μM)	N/A	-0.1 μM and 1.0 μM Ful increased Runx2 and OCN expression and mineralization in parallel with ROS scavenging ability.
